# Supplementary material for: Neutrophil Extracellular Traps in Airway Diseases: Pathological Roles and Therapeutic Implications
Source: Int J Mol Sci. 2023 Mar 6;24(5):5034. doi: 10.3390/ijms24055034 (PMC10003347; doi:10.3390/ijms24055034)
Supplement: Supplementary file 1 [file ijms-24-05034-s001.zip › ijms-2232083-supplementary.pdf]

PRISMA 2020 flow diagram for new systematic reviews which included searches of databases and registers only

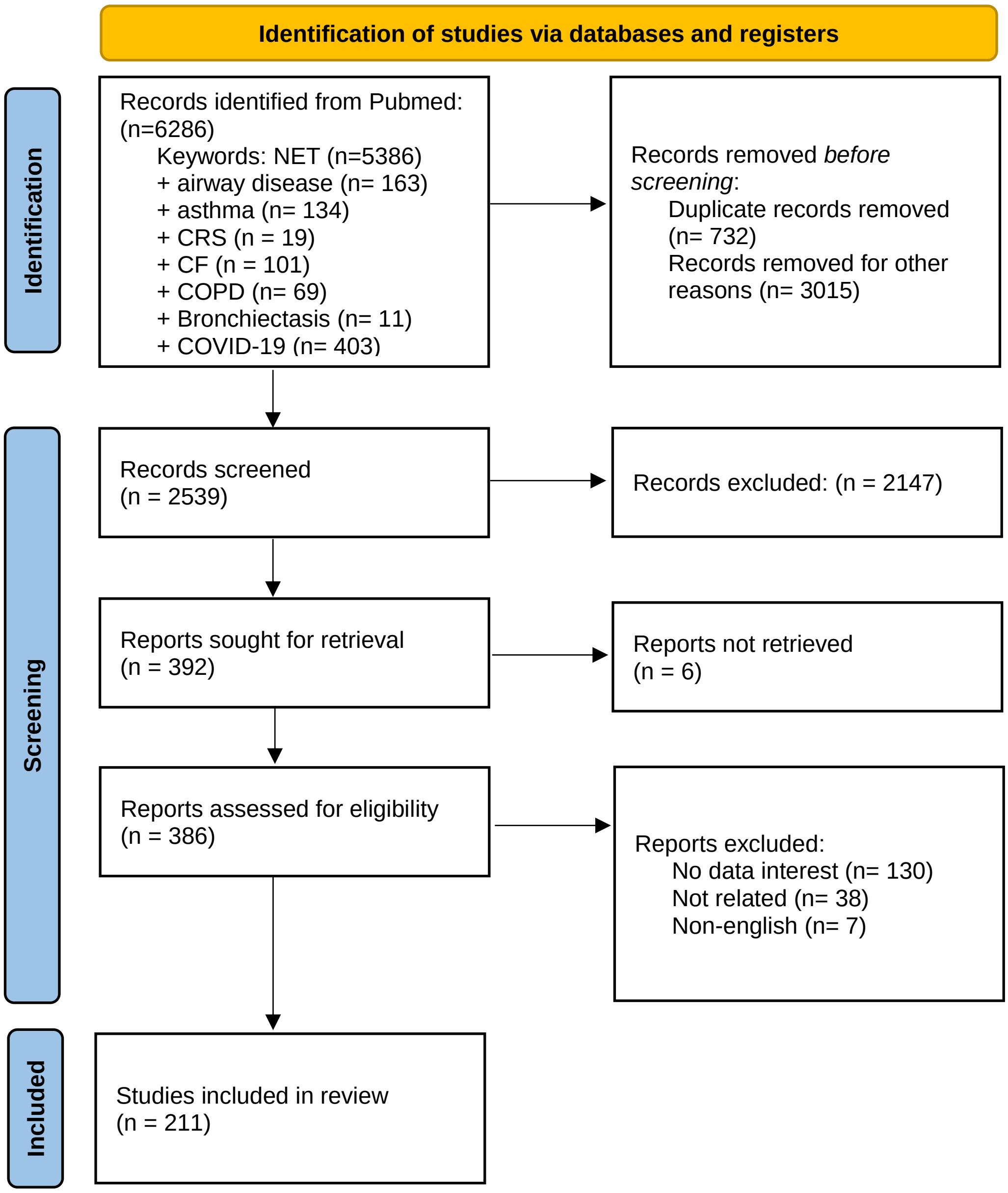

From: Page MJ, McKenzie JE, Bossuyt PM, Boutron I, Hoffmann TC, Mulrow CD, et al. The PRISMA 2020 statement: an updated guideline for reporting systematic reviews. BMJ 2021;372:n71. doi: 10.1136/bmj.n71

For more information, visit: http://www.prisma-statement.org/

Supplementary Figure S1. A flowchart summarized the study selection process in line with the PRISMA 2020 statement.
